# A systematic review and meta-analysis of serum cholesterol and triglyceride levels in patients with Parkinson’s disease

**DOI:** 10.1186/s12944-020-01284-w

**Published:** 2020-05-19

**Authors:** Xiaoxue Fu, Yu Wang, Xiaofeng He, Hongyu Li, Hong Liu, Xiangyang Zhang

**Affiliations:** 1 Heping Hospital Affiliated to Changzhi Medical College, Changzhi, Shanxi Province China; 2 Department of Neurology, Tianjin Medical University General Hospital, Tianjin, China; 3 Department of Science and Education, Heping Hospital Affiliated to Changzhi Medical College, Changzhi city, Shanxi P.R. China; 4 CAS Key Laboratory of Mental Health, Institute of Psychology, Chinese Academy of Sciences, 16 Lincui Road, Chaoyang District, Beijing, 100101 China; 5 Department of Psychology, University of Chinese Academy of Sciences, 16 Lincui Road, Chaoyang District, Beijing, 100101 China

**Keywords:** Meta-analysis, Systematic review, Parkinson’s disease, Triglyceride, Low density lipoprotein cholesterol, Total cholesterol

## Abstract

**Objectives:**

Numerous studies have reported that lipid metabolic abnormalities may play an important role in the development of Parkinson’s disease (PD), with mixed results. This meta-analysis aims to systematically assess the relationship between serum cholesterol or triglyceride and the PD risk and to further determine the role of dyslipidemia in potential predictive value.

**Methods:**

This research systematically consulted and screened observational studies to evaluate the association of serum lipids with the risk of PD as of April 01, 2020 based on the inclusion and exclusion criteria. Two researchers screened and extracted the data independently. Then this article summarized the characteristics of all clinical studies and collected the corresponding data to perform pooled and sensitivity analyses. The meta-analysis was performed by using the RevMan 5.3 software after data extraction, quality assessment and analysis of publication bias.

**Results:**

Twenty-one related studies (13 case-control and 8 cohort studies) were selected with a total of 980,180 subjects, including 11,188 PD patients. Meta-analysis showed that higher levels of serum triglyceride (S-TG) [standard mean different (SMD) = − 0.26 (95% confidence interval (CI): − 0.39 to − 0.13, *p*<0.00001), relative risk (RR) = 0.67 (95% CI: 0.60 to 0.75, *p*<0.00001)] could be considered as protective factors for the pathogenesis of PD. However, there was no significant association between serum high density lipoprotein cholesterol (S-HDL) and the risk of PD. Meanwhile, serum low density lipoprotein cholesterol (S-LDL) [SMD = -0.26 (95% CI: − 0.43 to − 0.07, *p* = 0.006), RR = 0.76 (95% CI: 0.59 to 0.97, *p* = 0.03)] and serum total cholesterol (S-TC) levels [SMD = -0.21 (95% CI: − 0.33 to − 0.10, *p* = 0.0002), RR = 0.86 (95% CI: 0.77 to 0.97, *p* = 0.01)] were negatively associated with PD risk.

**Conclusions:**

This systematic review suggests that elevated serum levels of TG, LDL and TC may be protective factors for the pathogenesis of PD. Further longitudinal and well-designed prospective studies with a large sample size are needed to confirm the findings in this meta-analysis.

## Introduction

Parkinson’s disease (PD) is a neurodegenerative disease, with a total of 5 million cases worldwide. With the aging of the population, PD cases are expected to double in the next two decades [1]. It is generally believed that the degeneration of dopamine-containing neurons in the mesencephalic substantia nigra pars compacta (SNc) may lead to PD [2, 3]. The pathogenesis of PD is complex, although many mechanisms such as environmental agents, genetic susceptibility and other possible factors have been discovered. Over the past several years, some findings have shown that changes in brain cholesterol homeostasis are associated with Alzheimer’s disease (AD), Huntington’s disease (HD) and other neurological diseases [4]. Numerous studies have shown that lipid metabolism plays an important role in the development of PD, but authors draw two opposite conclusions. For example, one study found a positive correlation between cholesterol intake and PD [5]. Accelerated loss of dopaminergic neurons may be the result of high-fat diets caused by neurotoxins in PD [6]. However, another prospective study [7] showed that high cholesterol intake was associated with a reduced risk of PD. Studies have shown that higher serum cholesterol concentrations are beneficial for slowing clinical progression and significantly reducing the risk of PD, while lower LDL concentrations may increase the incidence of PD [8, 9]. Therefore, lipids are also considered to be a marker of PD severity [10]. It has been suggested that a significant reduction in cholesterol and triglyceride biosynthesis in PD patients may explain this link [11]. However, some studies have confirmed that the neuroprotective effect of high levels of cholesterol may reduce and delay the onset of PD [12].

Several studies have evaluated the correlation between cholesterol or TG and PD risk, but the correlation is controversial and uncertain. In a previous meta analysis, Gudala et al. [13] searched for observational studies investigating the relationship between serum cholesterol and PD (published as of January 2013). Eight related studies were identified (4 case-control and 4 cohort studies). A combined analysis of eight studies with significant heterogeneity found no significant association between serum cholesterol and risk of PD (RR 0.87, 95% CI 0.67–1.13; *p* = 0.41). Subgroups were studied for type of study (case-control and cohort studies), gender and quality assessment results, but there was no evidence of heterogeneity in each subgroup.

To clarify the existing epidemiological evidence and analyze the relationship between serum cholesterol or TG levels and PD risk, this research systematically reviewed the literature again and performed a meta analysis. Compared to a previous meta analysis [13] this systematic review included more recent studies, with more reliable results.

## Materials and methods

### Search strategy

This research carried out a systematic search in two electronic databases: EMBASE and PubMed dating from 1947 to April 01, 2020. The search keywords or MESH terms were: “Cholesterol”, “Hypercholesterolemia”, “Triacylglycerol”, “Cholesterol, LDL”, “Cholesterol, HDL”, “Cholesterol, VLDL” and “Parkinson’s Disease”. PubMed was used to conduct a primary search and the identical terms were applied to search in other databases. Two researchers archived all documents independently. The search was limited to the published articles written in English and Chinese. Two authors (H.L. and X.X.F.) independently screened the titles and abstracts of all search results and then excluded publications that did not meet the criteria. At the same time, the full text of the related articles was obtained. Different opinions were discussed with the third review author (Y.W.) until an agreement was reached.

### Eligibility criteria and exclusion criteria

In current analysis, this research included the articles meeting the following criteria: (i) observational (cohort or case-control) studies; (ii) measurement of serum cholesterol, triglyceride or hypercholesterolemia as a main variable or covariate; (iii) analysis of PD as a dependent variable.

The exclusion criteria for screening studies included: (i) published only as abstract; (ii) case reports, reviews or clinical guidelines; (iii) did not assess PD at baseline; (iv) studies without effect data or valid data that were unable to be estimated and calculated.

### Data extraction and methodological quality

Data extraction was conducted in eligible studies, including: (i) the name of the first author, country, year of study, publication time, sex and basic information of participants; (ii) methods for assessing serum lipids and PD; (iii) criteria for selecting the healthy controls and the potential confounding factors; (iv) relevant statistics describing the relationship between serum lipids and PD. All of the above data were extracted independently by two authors (H.L. and X.X.F.).

This article used the New-castle Ottawa Scales (NOS) to independently assess the methodological quality of case-control and cohort studies that met the eligibility criteria by the two review authors (H.L, X.X.F). The NOS is divided into three parts: selection (total 4 points), comparability (total 2 points) and exposure or outcome (total 3 points). Therefore, 9 points are considered as the highest quality, 7 8 points as medium quality and ≤ 6 points as low quality. Disagreements were resolved by reevaluating the studies and discussing it with the third author (Y.W.).

### Statistical analysis

This research extracted continuous variables as SMD and dichotomous variables as RR or OR from included studies. Because the risk of PD is low, this research used RR instead of odds ratio (OR). When HR was reported, this research recommended that it is similar to the effect measurement in other studies. Statistical analysis was carried out, with 95% CIs as well as Q test and I square statistic to assess the heterogeneity between trials [14]. If significant heterogeneity was observed (I^2^ ≥ 50% and/or *p* < 0.1), the random-effect model was used to calculate the pooled estimates. Otherwise, the fixed-effect model was used to pool studies with low heterogeneity (I^2^ < 50% and/or *p* > 0.1) [15]. This research used the funnel plots, Egger’s statistical test to evaluate the publication bias for the analyses (these will not be calculated when fewer than 10 studies are analyzed as recommended by the Cochrane Collaboration) [16]. The stability of all results was evaluated by one-way sensitivity analysis [17]. In order to determine the source of the heterogeneity and examine the effects of potential factors, subgroup and meta-regression analyses were performed. All data was analyzed by using Stata version 14 and Review Manager 5.3.

## Results

### Search results

**Fig. 1:**
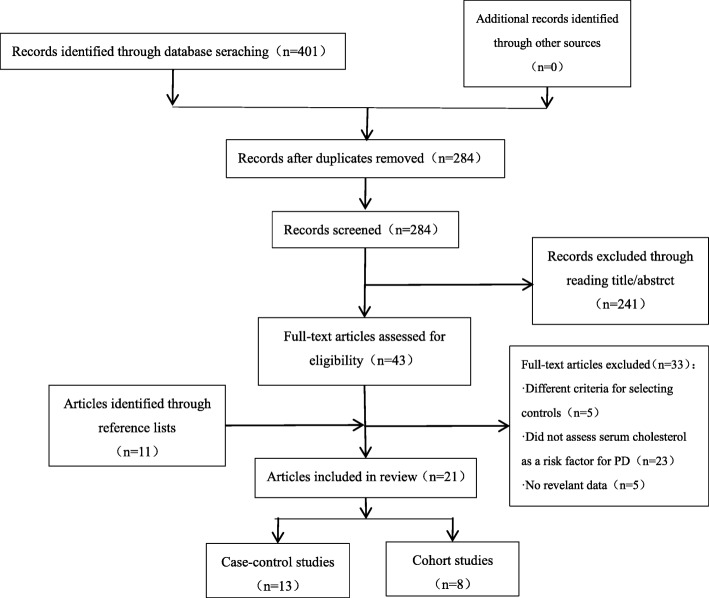
Flow diagram of study selection for the meta-analysis

These searches returned 284 records, of which 241 were excluded as irrelevant based on the reading of the title and abstract. The remaining 43 articles and 11 studies identified through the list of references were read in full by the two authors independently. Thirty-three studies were excluded because the criteria for selection of controls (*n* = 5) were different, no serum lipids were used as variables or covariates in analysis (*n* = 23) and there were no relevant data (*n* = 5). As a result, 21 studies met these requirements and were included in this analysis. Figure 1 shows a more detailed search process**.**

### Study characteristics and results of the quality assessment

Twenty-one eligible observational studies were selected, including 13 case-control [10, 11, 18–28] and 8 cohort studies [9, 29–35]. Thirteen case-control studies included 5529 PD patients and 6176 controls. Eight cohort studies included 968,475 subjects, with 5659 PD cases. Among these studies, 9 of the studies were conducted in Europe [9, 18, 22, 25, 26, 31–33, 35], seven in North America [11, 19, 24, 28–30, 34], and five in Asia [10, 20, 21, 23, 27]. Other details of the baseline data are shown in Tables 1 and 2.

**Table 1:** Characteristics of case-control studies

Author, Year	PD/control	Country	Mean PD duration	Mean H&Y scale	Assessment of PD	Assessment of status of S-C and S-TG
Becker 2008 [18]	3637/3637	UK	NA	NA	UKPD criteria/H&Y stage/UPDRS	Medical & Laboratories records
Du 2012 [19]	40/29	USA	4.2	1.5	UPDRS	Laboratories records
Guo 2015 [10]	555/555	China	3.8	2.5	UKPD criteria/H&Y stage/UPDRS	Laboratories records
Huang 2007 [11]	124/110	USA	4.2	NA	UPDRS	Medical & Laboratories records
Ikeda 2011 [20]	119/120	Japan	6.9	3.2	UKPD criteria	Laboratories records
Kim 2017 [21]	104/52	Korea	3.7	2.5	H&Y stage/UPDRS	Laboratories records
Kirbas 2014 [22]	42/40	Turkey	NA	NA	PDBBC/H&Y stage	Laboratories records
Miyake 2010 [23]	249/368	Japan	NA	NA	UKPD criteria	Using diet history questionnaire (DHQ)
Savica 2012 [24]	196/196	USA	NA	NA	H&Y stage/UPDRS	Laboratories records
Scigliano 2006 [25]	178/533	Italy	1.3	≈2	UKPD criteria/H&Y stage/UPDRS	Laboratories records
Vikdahl 2015 [26]	84/336	Sweden	NA	NA	UKPD criteria	Laboratories records
Wei 2013 [27]	110/130	China	NA	2.2	Met the Calne’s criteria of clinically definitive PD	Laboratories records
Zhang 2017 [28]	91/70	USA	NA	NA	MMSE	Laboratories records

Abbreviations: *UKPD* United Kingdom PD Society Brain Bank, *MMSE* Mini-Mental State Examination, *H&Y* Hoehn and Yahr scale, *UPDRS* Unified Parkinson’s Disease Rating Scale, *PDBBC* Parkinson’s Disease Brain Bank Criteria, *NA* not available

**Table 2:** Characteristics of cohort studies

Author, Year	Country	Size of cohort (M/F)	PD cases (M/F)	Age at baseline (Y)	Study period (starting-ending year)	Assessment of PD	Assessment of status of S-C and S-TG
Simon 2010 [29]	USA	171,879 (50,833/121046)	530 (NA)	30–75	22.9 (1976–2000)	Parkinsonian signs	Self reported
Huang 2008 [30]	USA	3223 (3223/0)	41 (41/0)	71–79	3 (1991–1993)	Medical records & neurologic examination	Laboratories records
Benn 2017 [31]	Danish	111,194 (49,884/61310)	460 (NA)	46–66	39 (1976–2014)	Medical records	Laboratories records
Saaksjarvi 2015 [32]	Finland	6641 (3102/3539)	89 (49/40)	30–79	30 (1978–2007)	Datebase	Laboratories records
de Lau 2006 [9]	Netherlands	6465 (2654/3811)	87 (46/41)	≥55	9.4 (1990–2004)	Parkinsonian signs & neurologic examination	Laboratories records
Hu 2008 [33]	Finland	50,926 (24,773/26153)	625 (321/304)	25–64	18.1 (1972–1997)	Medical records & neurologic examination	Laboratories records
Grandnetti 1994 [34]	USA	8006 (8006/0)	58 (NA)	71–93	26 (1965–1991)	Medical records	Laboratories records
Fang 2019 [35]	Sweden	610,141 (313,044/297097)	3769 (NA)	15–77	26 (1985–2011)	Medical records	Laboratories records

Abbreviations: *M* males, *F* females, *NA* not available, *Y* years

The Newcastle-Ottawa Scale (NOS) scores for these studies ranged from 7 to 9. All studies were considered to be of moderate to high quality (≥7).

### Pooled meta-analysis

**Fig. 2:**
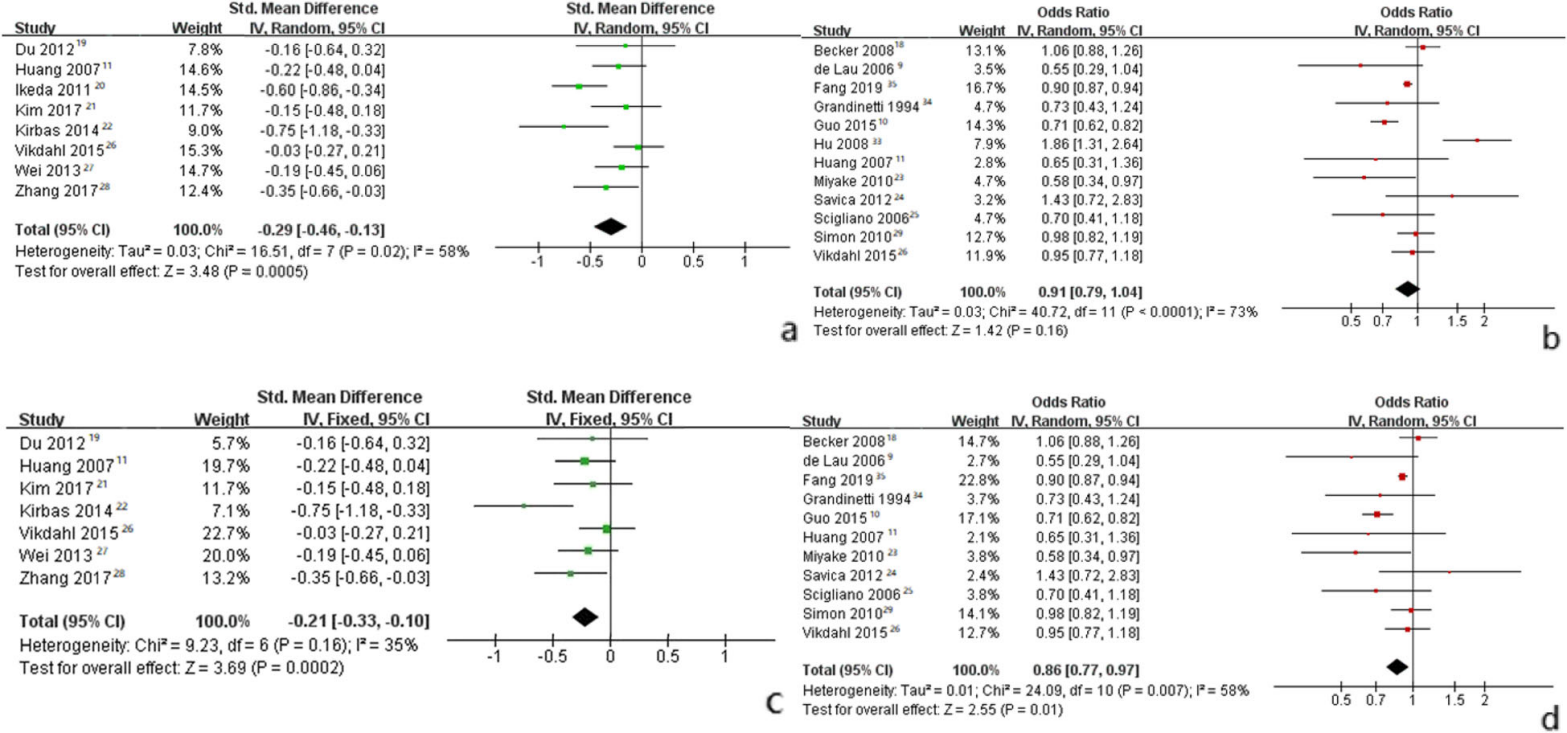
Forest plots for pooled standard weighted mean difference (**a**) or odds ratios (**b**) and the corresponding 95% confidence intervals (CIs) of studies assessing the association between S-TC levels and risk of Parkinson’s disease. Forest plots pooled standard weighted mean difference (**c**) or odds ratios (**d**) for meta-analysis after excluding the study by Ikeda et al. [20] or Hu et al. [33]

Random-effect meta-analysis of continuous data showed that the level of S-TC in patients with PD was significantly lower than that in controls [SMD = -0.29 (95% CI*:* − 0.46 to − 0.13, *p* = 0.0005)] (Fig. 2a). However, random-effect meta-analysis of dichotomous data between S-TC and PD did not show significant results [RR = 0.91 (95% CI: 0.79 to 1.04, *p* = 0.16)] (Fig. 2b).

**Fig. 3:**
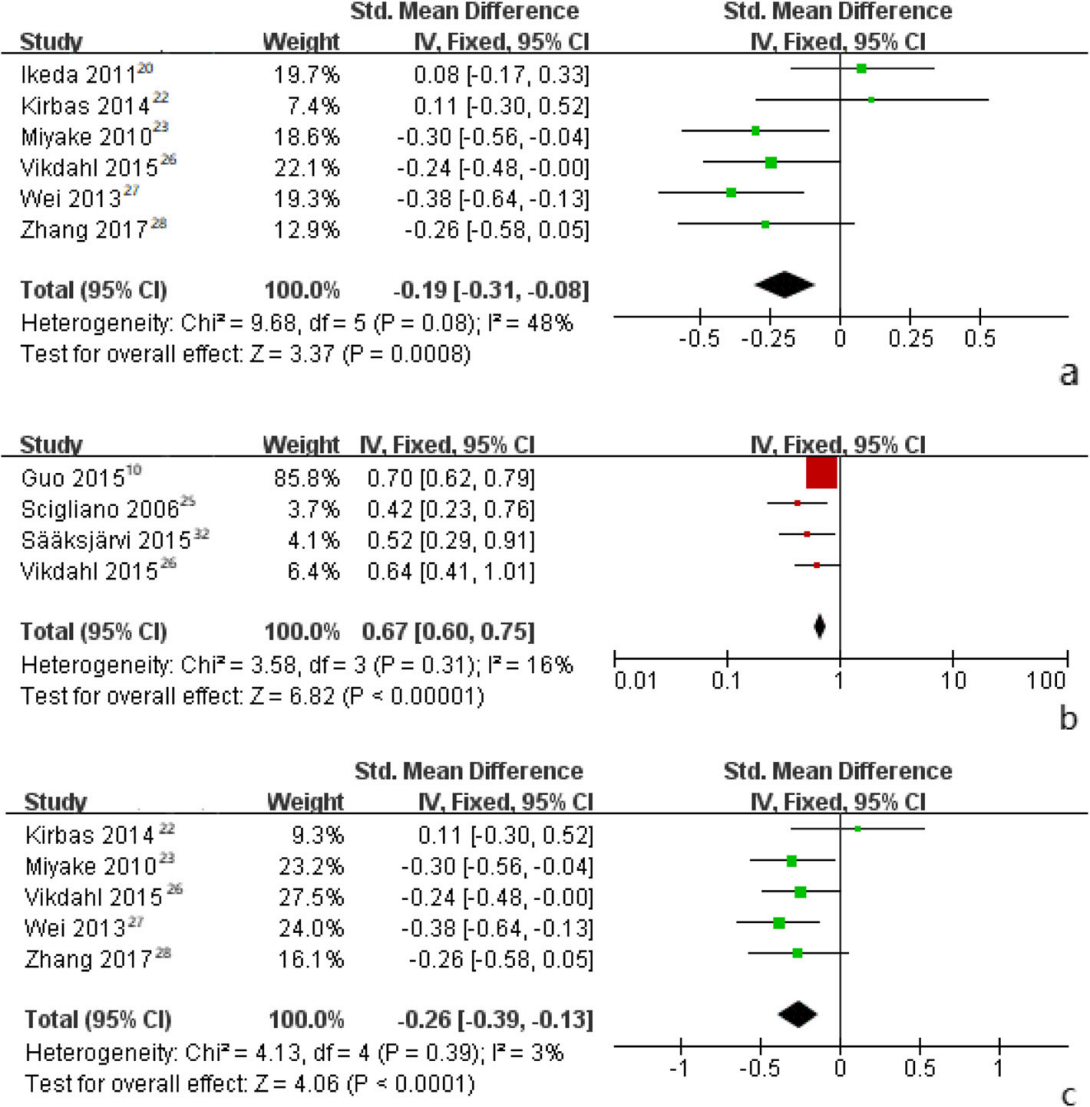
Forest plots for pooled standard weighted mean difference (**a**) or odds ratios (**b**) and the corresponding 95% confidence intervals (CIs) of studies assessing the association between S-TG levels and risk of Parkinson’s disease. Forest plots pooled standard weighted mean difference (**c**) for meta-analysis after excluding the study by Ikeda et al. [20]

Fixed-effect meta-analysis of continuous and dichotomous data showed that compared to controls, PD patients had significantly lower levels of S-TG [SMD = -0.19 (95% CI: − 0.31 to − 0.08, *p* = 0.0008), RR = 0.67 (95% CI: 0.60 to 0.75, *p*<0.00001)] (Fig. 3a, b), and LDL [SMD = -0.31 (95% CI: − 0.49 to 0.14, *p* = 0.0005), RR = 0.76 (95% CI: 0.59 to 0.97, *p* = 0.03)]. Furthermore, levels of S-HDL were not significantly different between PD patients and controls (Table 3).

**Table 3:** Summary of Comparative Outcomes for serum lipid Levels

Data type	Item	No. of studies	PD/Control	Main effect			Heterogeneity				Publication bias	
				Hedges g(95%CI)	z score	*p* value	Q statistic	df	p value	I^2^ statistic	Egger intercept	p value
Continuous	TC	8	724/889	-0.29 (−0.46, −0.13)	3.48	0.0005	16.51	7	0.02	58%	−1.9330	0.483
	adjustion	7	605/769	−0.21 (− 0.33, − 0.10)	3.69	0.0002	9.23	6	0.16	35%	–	–
	TG	6	542/865	−0.19 (− 0.31, − 0.08)	3.37	0.0008	9.68	5	0.08	48%	2.8479	0.472
	adjustion	5	423/745	−0.26 (−0.39, − 0.13)	4.06	<0.0001	4.13	4	0.38	3%	–	–
	LDL	6	536/501	−0.31 (−0.49,-0.14)	3.48	0.0005	9.57	5	0.09	48%	−0.9563	0.974
	adjustion	5	417/381	−0.26 (− 0.43, − 0.07)	2.74	0.006	6.59	4	0.16	39%	–	–
	HDL	5	496/472	0.08 (−0.04, 0.21)	1.28	0.20	1.94	4	0.75	0%	0.4329	0.851
Dichotomous	TC	12	–	0.91 (0.79, 1.04)	1.42	0.16	40.72	11	<0.0001	73%	−0.3306	0.965
	adjustion	11	–	0.86 (0.77, 0.97)	2.55	0.01	24.09	10	0.007	58%	–	–
	TG	4	–	0.67 (0.60,0.75)	6.82	<0.00001	3.58	3	0.31	16%	−1.3695	0.107
	LDL	4	–	0.76 (0.59,0.97)	2.23	0.03	16.89	3	0.0007	82%	−2.3686	0.205

Abbreviations: *TC* total cholesterol, *TG* triglycerides, *LDL* low density lipoprotein cholesterol, *HDL* high density lipoprotein cholesterol, *C* cholesterol, *S* serum

### Investigation of heterogeneity

**Fig. 4:**
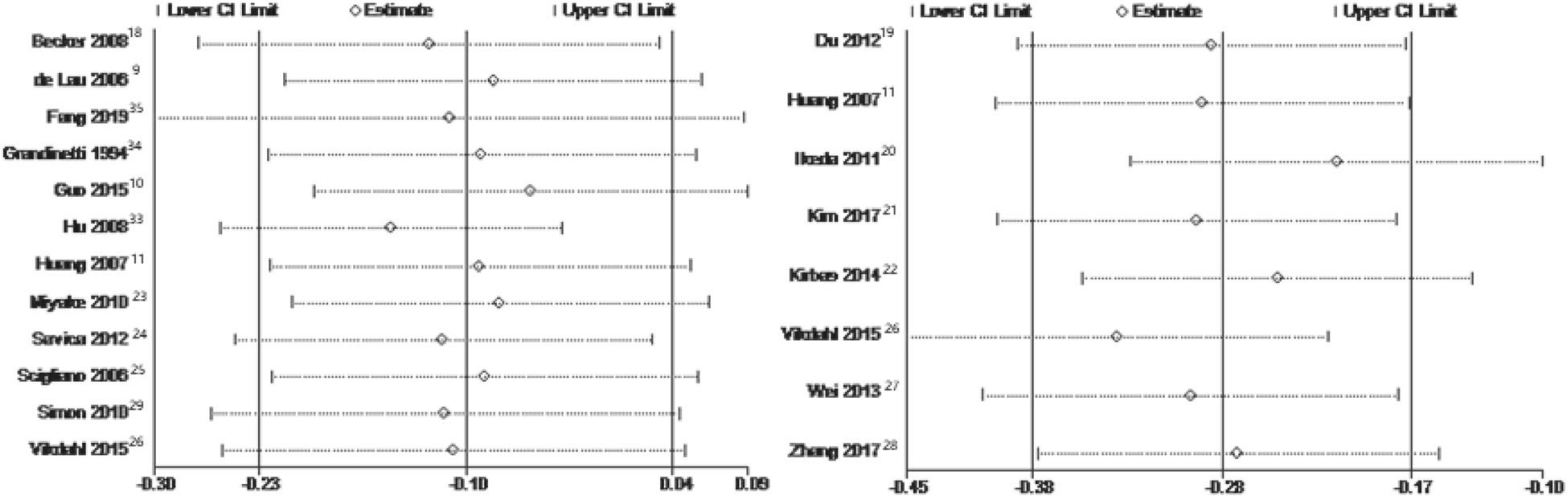
Sensitivity analyses for pooled standard weighted mean difference and odds ratios of studies assessing the association between S-TC levels and risk of Parkinson’s disease using a leave-one-out strategy

The pooled effect size of 12 S-TC dichotomous data between PD patients and controls were quite heterogeneous between studies (I^2^ = 73%, *p* for heterogeneity<0.0001). Therefore, This research used leave-one-out strategy for sensitivity analyses (Fig. 4) to assess the source of heterogeneity. The study by Hu et al. [33] mainly included subjects aged 25–64 years, showing a positive association between S-TC and the risk of PD. Studies from other countries included relatively older subjects than this study. After the removal of study by Hu et al. [33], the between-study heterogeneity was significantly decreased (I^2^ = 58%, *p* = 0.007), and the pooled RR became 0.86 (95% CI: 0.77 to 0.97, *p* = 0.01) (Fig. 2d).

The results of combining 8 continuous S-TC levels indicated that S-TC levels were significantly decreased in PD patients. However, there was considerable heterogeneity (I^2^ = 58%, *p* for heterogeneity = 0.02) between studies. Therefore, this research also performed sensitivity analyses (Fig. 4) to evaluate the source of heterogeneity. In the study of Ikeda et al. [20], the mean Hoehn and Yahr (H&Y) stage (standard deviation, SD) was 3.2 (0.9), which means that PD patients had more severe symptoms in this study. Other studies included subjects with a relatively lower mean H&Y score and average PD duration or subjects with the first or most recent diagnosis. After excluding this study, the between-study heterogeneity was much lower (I^2^ = 35%, *p* = 0.16), and SMD became − 0.21 (95% CI: − 0.33 to − 0.10, *p* = 0.0002) (Fig. 2c).

Similarly, for the pooled results from all continuous data of S-TG levels except for the data of Ikeda et al. [20], heterogeneity turned to be much lower (I^2^ = 3%, *p* = 0.39) and SMD became − 0.26 (95% CI: − 0.39 to − 0.13, *p*<0.0001) (Fig. 3c). Combining the findings from all continuous data of LDL levels except for the data of Ikeda et al. [20], heterogeneity turned to be much lower (I^2^ = 39%, *p* = 0.16) and SMD became − 0.26 (95% CI: − 0.45 to − 0.07, *p* = 0.006). There were still significant differences after adjusting the studies (Table 3).

Subgroup and meta-regression analyses were also performed on gender, race and different techniques to measure lipids, but there was no evidence of heterogeneity in each subgroup. No publication bias was found in these analyses because an asymmetric distribution was shown in each funnel plot. Both the Egger’s tests produced the same results (Tables 3). When the number of studies is small, these studies are much under-powered. Therefore, as part of the study, this research did not conduct statistical tests on publication bias.

## Discussion

This review aimed to systematically assess the link between serum cholesterol or triglyceride and the risk of PD. Twenty-one relevant studies (13 case-control and 8 cohort studies) were included involving a total of 980,180 subjects, including 11,188 PD patients. According to the meta-analysis, this research observed that higher level of S-TG was statistically useful as protective factor for the onset of PD, but there was no significant correlation between S-HDL and the risk of PD. Meanwhile, it was found that the level S-TC and S-LDL were negatively correlated with the risk of PD. Considering that there is still a high heterogeneity between studies after excluding the studies with the higher heterogeneity, this research need to be cautious to conclude that S-TC and S-LDL may be associated with a decreased risk of PD. More studies are needed before drawing an affirmative conclusion.

### Cholesterol and PD

Most of the cholesterol in the brain is produced by cerebral astrocytes. Cholesterol plays a key role in either the synthesis of myelin by intracerebral oligodendrocytes or proper maintenance of synaptic function. Depletion of cholesterol in neurons may lead to degeneration of dendritic spine and synapse, impairment of synaptic vesicle exocytosis and disorders of neuronal activity and neurotransmission [36]. To some extent, the quantitative abnormalities of serum lipids were correlated with metabolic dysfunction. A large body of evidence has also indicated that defects in cholesterol metabolism may cause a variety of central nervous system (CNS) diseases such as HD, AD and PD [37–39].

Many putative mechanisms support an inverse correlation between serum lipids and the risk of PD. Coenzyme Q10 is known to be an important electron acceptor of complexes I and II existed in mitochondria and is also an effective antioxidant [29, 31]. Serum levels of coenzyme Q10 are largely dependent on serum cholesterol levels. Some studies have shown a negative correlation between cholesterol levels and PD, which may be due to the neuroprotective effect of coenzyme Q10 that is reduced with the decrease of serum cholesterol [12]. Animal experiments have also shown that coenzyme Q10 is closely related to the pathogenesis of PD due to its powerful neuroprotective effects and its function of decreasing oxidative stress. Also, it can delay the depletion of dopamine [38]. On the other hand, many mechanisms support the hypothesis that higher serum cholesterol levels increase the risk of PD. In the pathogenesis of PD, oxidative stress, inflammatory reaction, mitochondrial dysfunctions, and excitotoxicity synergistically contribute to its complex molecular mechanisms [40, 41].

Hypercholesterolemia or oxysterols may cause gliosis in the brain, which release pro-inflammatory mediators and other oxidative changes [42], resulting in oxidative stress, dysfunction of mitochondria or neuroinflammation [43, 44]. A study has shown that serum lipids including cholesterol are associated with the accumulation of toxic α-synuclein and neuromelanin [45]. As a product of cholesterol oxidation, oxysterols will induce the aggregation of α-synuclein, which then leads to apoptosis of dopaminergic cells [46, 47].

Recent studies have shown that the use of statins may reduce the aggregation of α-synuclein in PD patients by lowering plasma cholesterol [48]. In animal or cell experiments, lipid-lowering agents are believed to have potential therapeutic implications in PD because of their anti-inflammatory, anti-oxidant, and antiplatelet effects [49]. Many studies have also shown that the use of lipid-lowering drugs was more or less effective in PD patients [50–52].

### Triglyceride and PD

This study showed that lower concentrations of serum triglyceride were a potential risk of PD. Many hypothetical mechanisms attempt to explain this result. Studies have shown that autonomic failure might be accompanied by the development of the neurodegenerative processes [53, 54]. Similarly, sympathetic activity was also reduced in PD patients [55], and TG levels were significantly declined due to reduced production of catecholamine and cortisol. Since non-motor symptoms have preceded motor symptoms for decades [56], lower levels of cholesterol may be the result of autonomic disorders or extensive sympathetic denervation, earlier than the clinical diagnosis of PD [25, 57]. In addition, it has been reported that dopamine converted by levodopa could exert its peripheral effects and reduce S-TG levels, and PD patients taking levodopa as a medicine are more likely to reduce S-TG than other PD patients [58, 59].

Similarly, nutrition also contributes to lipid profile. Because the competitive effects between levodopa and food proteins occur in transportation across the blood-brain barrier and intestinal absorption, excessive protein-rich diet is not recommended for PD patients who were treated with levodopa. Studies have shown that PD patients prefer to consume carbohydrates rather than milk and its derivatives [60]. The intake of polyunsaturated fatty acids is also lower [61]. Food intake may also be affected by reduced activities or dyskinesia. All of the dietary habits listed above may affect serum lipids more or less.

### Confounding factors

The actual relationship may be masked by various confounding factors: (i) Serum lipids may decline during the development of other chronic diseases, such as AD and some neurodegenerative diseases [62–64]. (ii) Patients with a prescription of cholesterol-lowering agents or statins in their studies; (iii) Special dietary habits and dyskinesia of PD patients; (iv) Smoking, BMI, coffee consumption and other factors that may influence the risk of PD; (v) Environmental and genetic backgrounds may play an important role to some extent. In this study, this research fully considered the bias caused by these confounding factors. This article preferred the adjusted data in this analysis, but where there was no adjusted data, this research chose the unadjusted data. Meanwhile, the above factors should be fully considered in the following experimental design.

## Conclusion

According to available epidemiological evidence, higher levels of S-TG may be protective factors for PD onset. Meanwhile, it is prudent to draw a conclusion that S-TC and S-LDL may be associated with a decreased risk of PD. It is of great significance to explore the potential links and molecular mechanism between them to determine whether the changes in serum lipid metabolism are causal or consequence. However, most studies, as case-control studies rather than prospective studies, could not make causal inference regarding the risk of PD and serum lipids. Further longitudinal and well-designed prospective studies with a large sample size are needed to investigate the association between the PD severity or clinical progression and lipid levels. With the deepening of researches, effective intervention measures for cholesterol and its metabolites are also of great clinical significance for delaying the development of Parkinson’s disease and finding new therapeutic targets.

## Data Availability

The data analyzed during the present study are available from the corresponding author on reasonable request.
